# Transparent Cellulose-Based Films Prepared from Used Disposable Paper Cups via an Ionic Liquid

**DOI:** 10.3390/polym13234209

**Published:** 2021-12-01

**Authors:** Zhen Xu, Qiwen Zhou, Lixiang Wang, Guangmei Xia, Xingxiang Ji, Jinming Zhang, Jun Zhang, Haq Nawaz, Jie Wang, Jianfeng Peng

**Affiliations:** 1 State Key Laboratory of Biobased Material and Green Papermaking, Qilu University of Technology, Shandong Academy of Sciences, Jinan 250353, China; xz521953@qlu.edu.cn (Z.X.); biliqwzhou@163.com (Q.Z.); 15726110037@163.com (L.W.); WJ05111129@163.com (J.W.); pjf1966614429@163.com (J.P.); 2 Beijing National Laboratory for Molecular Sciences, CAS Key Laboratory of Engineering Plastics, Institute of Chemistry, Chinese Academy of Sciences (CAS), Beijing 100190, China; jzhang@iccas.ac.cn (J.Z.); haqnawaz@bjfu.edu.cn (H.N.)

**Keywords:** paper cups, recycling, cellulose, ionic liquid, cellulose-based films

## Abstract

Paper cups are widely employed in daily life with many advantages, but most of the used paper cups are incinerated or landfilled, due to the great challenge of separating the thin inner polyethylene (PE) coating, causing the waste of energy and the pollution of our environment. Therefore, recycling and converting the used paper cups into high-value materials is meaningful and important. In this work, transparent cellulose-based films were successfully prepared from the used paper cups via 1-allyl-3-methylimidazolium chloride ionic liquid after simple pretreatment. Additionally, the difference in properties and structures of cellulose-based films regenerated in different coagulation baths (water or ethanol) was also explored. It was found that the cellulose-based film possessed good thermal property and displayed better hydrophobicity than the traditional pure cellulose film. Moreover, they also demonstrated good mechanical property and the tensile strength of cellulose-based film regenerated in water can reach 31.5 Mpa, higher than those of cellulose-based film regenerated in ethanol (25.5 Mpa) and non-degradable polyethylene film (9–12 MPa), indicating their great potential as the packaging materials. Consequently, valorization of the low cost used paper cups and preparation of high-valve cellulose-based films were realized simultaneously by a facile and green process.

## 1. Introduction

Paper cups have many merits such as convenient carrying, low cost, water resistance, etc. and have been widely used in daily life and their yields increase dramatically year by year [1,2]. It is reported that China consumes over 10 billion disposable paper cups annually, while 50 billion paper cups are sold and consumed every year in the United States, indicating that many trees have been cut down to produce these paper cups and a large volume of carbon dioxide has been produced in this process [1,3]. Moreover, the used paper cups (UPCs) are always discarded as waste after use. In the UK, approximately 2.5 billion paper cups are consumed and 30,000 tons coffee paper cup waste are produced each year, while more than 7000 tons of non-recyclable UPCs waste are abandoned in Australia [1,3]. Consequently, most of the used paper cups are incinerated and landfilled at the end of their life, leading to serious environmental pollution and great waste of energy [1,2,4]. Therefore, it is urgent and important to develop environmentally friendly methods to recycle and reuse these used paper cups and even to add new high-valve to UPCs.

As the most abundant, renewable, biodegradable and eco-friendly biomaterial, cellulose has been widely used in papermaking, pharmaceutic adjuvant, food additives, pesticide, aerospace, and so on [5,6,7,8,9,10]. However, the high-grade dissolving-grade pulps including wood pulps and cotton liner pulps are still the major source for producing cellulose-based materials, making the raw materials scarce and limiting the exploitation and application of cellulose-based products [11,12,13]. In contrast, the domestic wastes which are produced extensively every year can be a good candidate as raw materials [14,15,16]. Generally, the paper cups are composed of 95 wt% high-quality cellulose paper board and 5 wt% inner polyethylene (PE) coating [1,4]. Therefore, the used paper cups can show promising feedbacks for cellulose.

Unfortunately, few investigations have been conducted to recycle and reuse UPCs in the last decades because the PE coating and the cellulose paper board are bonded tightly and it is difficult to separate them completely and economically, not to mention to fabricate cellulose-based materials from the UPCs [1,3,17,18]. It is found that UPCs can be recycled by physical, chemical, and biological methods [2,19,20]. By using the physical method, Mitchell et al. fabricated paper plastic composites (PPCs) by using shredded UPCs and polypropylene as the enhanced phase and matrix, respectively [19]. The mechanical property of PPCs was increased by adding the shredded UPCs, indicating that the UPCs can be reused as reinforcement in the composites. Karthika Arumugam et al. studied the degradation of the UPCs through vermicomposting, proving that earth worm and microbes (VCB) vermicomposting can short the UPCs degradation period and the degraded UPCs can be used as the fertilizer [2]. Zhao et al. and Chen et al. fabricated graphene sheets and carboxymethyl cellulose by using UPCs as raw materials [17,20,21]. Meanwhile, cellulose nanocrystals and cellulose nanofibers can also be obtained through a citric acid hydrolysis chemical method from the UPCs [18]. All these studies realized the recycling or reuse of the UPCs. However, converting the UPCs into new high-value products is rare. Furthermore, more works should be initiated to develop green and feasible methods for valorization of UPCs.

Cellulose is recalcitrant and cannot be dissolved in most organic solvents [22,23,24]. Nevertheless, it has been proved that cellulose can be dissolved efficiently in ionic liquids (ILs) [25,26,27]. Moreover, converting low-cost cellulose-contained wastes into high-value cellulose-based materials has been investigated for many years in our previous studies [12,13,16,25,28]. In this work, by using UPCs as raw materials, transparent cellulose-based films with excellent mechanical and thermal properties were successfully prepared through a green solvent ionic liquid 1-allyl-3-methylimidazolium chloride (AmimCl). Meanwhile, polarized optical microscopy (POM), Fourier transform infrared (FTIR), wide-angle X-ray diffraction (WAXD), ultraviolet and visible (UV-Vis) spectra, Thermogravimetric analysis (TGA), scanning electron microscopy (SEM), mechanical test, and contact angle test were used to investigate the structures and properties of the raw materials and cellulose-based films. Additionally, the influence of water and ethanol coagulation baths on the structures and properties of the regenerated cellulose-based materials was also explored.

## 2. Materials and Methods

### 2.1. Materials and Reagents

The 1-allyl-3-methylimidazole chloride (AmimCl) and the high purity cotton liner pulps (CPs) were kindly supplied by Shandong Henglian New Materials Co., Ltd. (Weifang, China), where the degree of polymerization of CPs is 530. Wood pulp disposable paper cups were purchased from Hangzhou Miuge Chemical Commodities Science & Technology Co., Ltd. (Hangzhou, China) and only used twice for drinks, and the mass contents of wood pulp and PE coating were 95 wt% and 5 wt%, respectively. Ethanol and sodium hypochlorite disinfection solution containing approximately 50 g/L available chlorine were purchased from Jinan Hengyou New Material Technology Co., Ltd. (Jinan, China) and used as received. Deionized water was self-produced by the ultra-pure water equipment.

### 2.2. Remove the Inner PE and Printing Pattern Coatings from the UPCs

The used disposable paper cups (UPCs) should be pretreated to remove impurities such as the inner PE and printing pattern coatings. Initially, UPCs were cut and immersed into the deionized water at 20 °C for 60 min and then PE coating was removed from the cellulose paper board. Subsequently, cellulose paper boards were treated with deionized water and sodium hypochlorite disinfection solution mixed solution at 60 °C for 15 min to get rid of the colors such as printing pattern coating, in which the mass ratio of the two liquids was 1:1 and the mass ratio between paper boards and aqueous sodium hypochloride solution (1:20). Then, the pretreated used paper cups (p-UPCs) were washed with distilled water for several times until a neutral pH was reached. Finally, the dried p-UPCs were shredded into pieces for further use. In the end, the weighing method was used to evaluate the recovery rate of cellulose paper board and its recovery rate was higher than 85%.

### 2.3. Dissolution of p-UPCs and Preparation of Cellulose-Based Films

**Figure 1:**
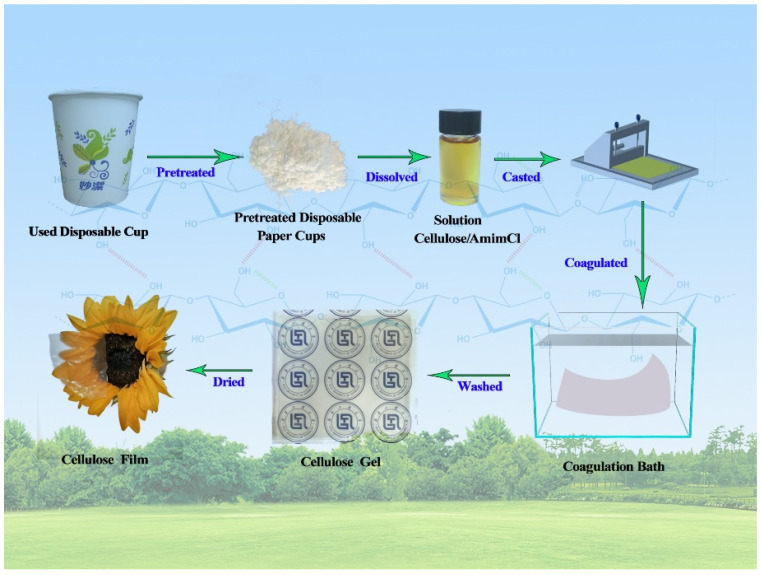
The procedure for the dissolution of used disposable paper cups and the fabrication of cellulose-based gels and films.

The procedure for the dissolution of pretreated used paper cups (p-UPCs) and the fabrication of cellulose-based films were briefly described in Figure 1. Firstly, 2 g of shredded p-UPCs and 98 g of AmimCl were added into a 250 mL three-necked flask, and the mixture was mechanically stirring at 80 °C for 180 min and a transparent solution was finally obtained. After that, the solution was cast onto a glass plate with a spreader to form 1000 um thick liquid film. Then, the liquid film was immediately transferred into water or ethanol coagulating bath to form cellulose-based gels by the sol-gel method. The coagulating bath was changed twice a day until no Cl- was detected by using silver nitrate titration and the last coagulating bath for cellulose-based gels were water and glycerol mixed solution (*v*/*v* = 95:5). Two kinds of transparent gels were obtained from the deionized water and ethanol coagulating bath and they were named as Gel-H and Gel-A, respectively. Finally, to get cellulose-based films (named as Film-H and Film-A), Gel-H and Gel-A were dried at 100 °C for 10 min in the Kessel paper dryer. The used ionic liquids can be recycled and reused as the solvent again, meaning that fabricating transparent cellulose-based films from used disposable paper cups via AmimCl ionic liquid solvent method is a relatively green process [15].

Meanwhile, by using the high-grade cotton liner pulp as raw material, the pure cellulose gel (Gel-C) and cellulose film (Film-C) was also prepared at the same conditions as described above for comparison.

### 2.4. Characterization

#### 2.4.1. The Degree of Polymerization (DP) of Cellulose in UPCs

Ubbelodhe viscometry was employed to measure the degree of polymerization (DP) of cellulose in UPCs, where cupriethylenediamine was used as the solvent. The Standard Test Method for Intrinsic Viscosity of Cellulose (ASTM D795-13) described the detailed information of this experiment. The degree of polymerization (DP) of cellulose in UPCs was about 288.

#### 2.4.2. Polarized Optical Microscopy of p-UPCs/AmimCl Solution

PM6000 polarizing microscope bought from Nanjing Jiangnan Yongxin Optical Co., Ltd. (Nanjing, China) was adopted to assess the dissolution process of p-UPCs in AmimCl. The clean cover slip and glass slide were used to sandwich the p-UPCs/AmimCl solution, and the sample of p-UPCs/AmimCl solution was taken every hour to observe the dissolution capability.

#### 2.4.3. Ultraviolet and Visible (UV-Vis) Spectra of the Cellulose-Based Films

The Ultraviolet spectrophotometer UV 2600 imported from Shimadzu of Japan was used to record the UV-Vis spectra of cellulose-based films, where the wavelength ranged from 400 to 800 nm.

#### 2.4.4. The Surface Hydrophilicity of the Cellulose-Based Films

The hydrophilicity of the PE film and cellulose-based films were characterized by OCA 50, which was purchase from Dataphysics of Stuttgart, Germany. The water contact angles of Film-H, Film-A, and Film-C were recorded, and five spots were recorded for each sample and the average value was reported.

#### 2.4.5. Wide-Angle X-ray Diffraction (WAXD) of the CPs, UPCs, P-UPCs, PE Film, and Cellulose-Based Films

The D8 AD-VANCE X-ray diffractometer bought from Bruker of Germany was adopted to record the X-ray diffraction patterns of the samples, where the 2θ span ranged from 5 to 60° and the scan speed was at 8°/min. CuKαRadiation (λ = 1.5406 Å), 40 kV, and 40 mA were employed.

#### 2.4.6. Fourier Transform Infrared (FTIR) Spectra of the CPs, UPCs, P-UPCs, PE Film, and Cellulose-Based Films

The attenuated total reflectance Fourier transform infrared spectrometer (ATR-FTIR ALPHA) purchased from the Bruker in Germany was used to study the chemical structure of samples. Germanium crystal was washed by using ethanol between samples and five spots were detected. The resolutions were 32 scans and 4 cm^−1^, and the results were analyzed by the OPUS software.

#### 2.4.7. Mechanical Testing of the Cellulose-Based Films

The TA.XT Plus C texture Analyzer purchased from StableMicroSystem in UK was employed to measure the tensile strength of the cellulose-based films. The width and length were 1 cm and 5 cm, respectively. The gauge length was maintained at 2 cm and the drawing speed was at 5.0 mm min^−1^. Seven sample strips were measured for Film-H and Film-A, and the average value was reported. The mechanical test was conducted by following the ASTM D-882 standard.

#### 2.4.8. Thermogravimetric Analysis (TGA) of the CPs, UPCs, P-UPCs, PE Film, and Cellulose-Based Films

The thermogravimetric analyzer (TA Q50) bought from the United States was used to investigate the thermal decomposition behavior of the regenerated cellulose-based films and the raw materials. TA had a ceramic pan inside the furnace and possessed a precision balance. About 5 mg of samples were put into the crucible pot, and the PE film and cellulose-based films should be cut into small pieces. Nitrogen atmosphere, a heating rate of 10 °C/min, and a span of 50 to 800 °C were set for all samples. 

#### 2.4.9. Morphology of the Cellulose-Based Films

The scanning electron microscopy (SEM) EM-30 Plus microscope imported from COXEM of Korea was used to characterize the morphology of the cellulose-based films. The cellulose-based films were quenched in the liquid nitrogen to get the cross section images of Film-H and Film-A. All cellulose-based films were sputter-coated with platinum before SEM test.

## 3. Results and Discussion

### 3.1. Pretreatment and Dissolution of Used Paper Cups

**Figure 2:**
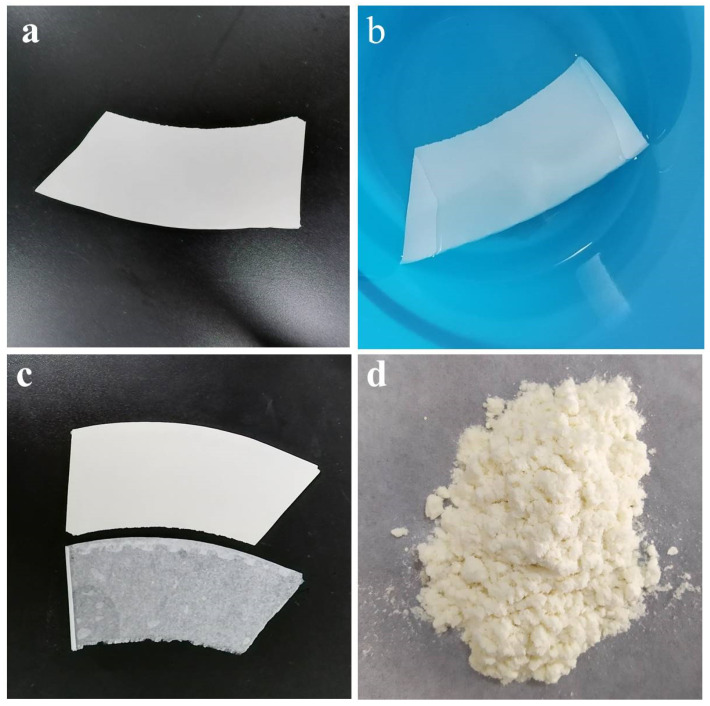
The digital pictures of (**a**) cut-up and used paper cups (UPCs), (**b**) used paper cups soaked in deionized water, (**c**) the inner thin PE film and the cellulose paper board, and (**d**) used paper pulp board after shredding (p-UPCs).

UPCs were pretreated firstly before dissolution and the digital pictures of the p-UPCs/AmimCl solution at different stages are displayed in Figure 2a–d. Firstly, the UPCs were cut and placed into deionized water for 60 min at 25 °C to wet UPCs thoroughly and loose the adhesion between cellulose paper board and PE coating (Figure 2a,b). Subsequently, the inner thin PE coating was separated from the cellulose paper board. Then, the PE film stuck with wood pulp and cellulose-based paper boards were obtained, respectively (Figure 2c). Next, cellulose paper boards were treated with deionized water and sodium hypochlorite disinfection mixed solution at 60 °C for 15 min to rid impurities such as printing pattern coating, in which the mass ratio of the two liquids was 1:1. Then, the pretreated used disposable paper cups (p-UPCs) were washed with distilled water for several times until a neutral pH was reached. Finally, the dried p-UPCs were shredded into pieces for further use (Figure 2d) and the p-UPCs became fluffy, which is good for the following dissolution.

**Figure 3:**
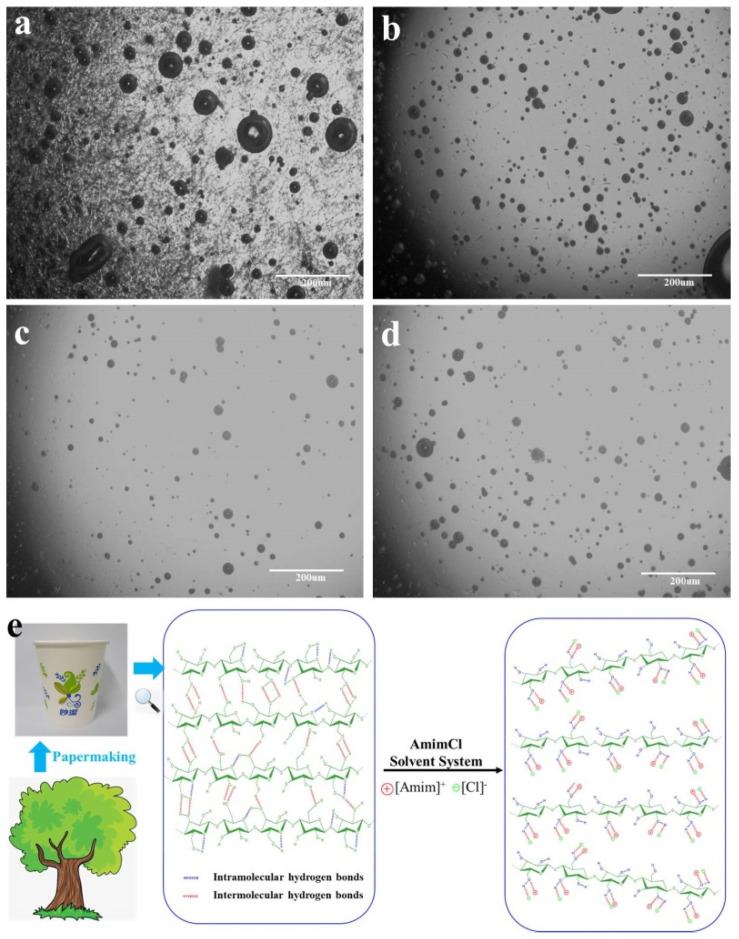
(**a**–**d**) POM micrographs of p-UPCs/AmimCl solution at 80 °C after (**a**) 0 h, (**b**) 1 h, (**c**) 2 h, and (**d**) 3 h; (**e**) the dissolution mechanism of p-UPCs cellulose in AmimCl solvent system.

Cellulose cannot be dissolved in most organic solvents and Rogers et al. [24] firstly reported that ionic liquids (ILs) show superior dissolving capacity for cellulose. Moreover, it has been found that ionic liquid AmimCl was one of the most effective solvents for cellulose [26,27]. In order to study the solubility of the p-UPCs in AmimCl, the POM of p-UPCs/AmimCl was recorded every one hour and the results are shown in Figure 3a–d. It is obvious that p-UPCs consist of a large number of microfibers whose diameters range from 10 to 50 μm (Figure 3a) and the microfibers are short after shredding. Initially, numerous short cellulose fibers were entangled and distributed randomly in AmimCl. However, the number of un-dissolved fibers decreased dramatically and only few shorter cellulose fibers were scattered in AmimCl after 1 h (Figure 3b). After 2 h, nearly all of the microfibers disappeared (Figure 3c) and the state of the solution at 3 h was similar to that of 2 h (Figure 3d), which indicated that most microfibers of the p-UPCs were dissolved completely after 3 h. Furthermore, the main component of p-UPCs is cellulose and the dissolution mechanism of cellulose in ionic liquid has been investigated and reported. It is proven that both cations and anions of ILs demonstrated a synergistic effect in the cellulose dissolution process and dissolution mechanism of the p-UPCs cellulose in AmimCl, as shown in Figure 3e [29,30]. Meanwhile, many bubbles were observed in the p-UPCs/AmimCl solution during the whole dissolve process due to the high viscosity of p-UPCs/AmimCl solution, and removing these bubbles is necessary before preparing these cellulose-based films. 

### 3.2. Transparency and Hydrophilicity of Cellulose-Based Gels and Films

**Figure 4:**
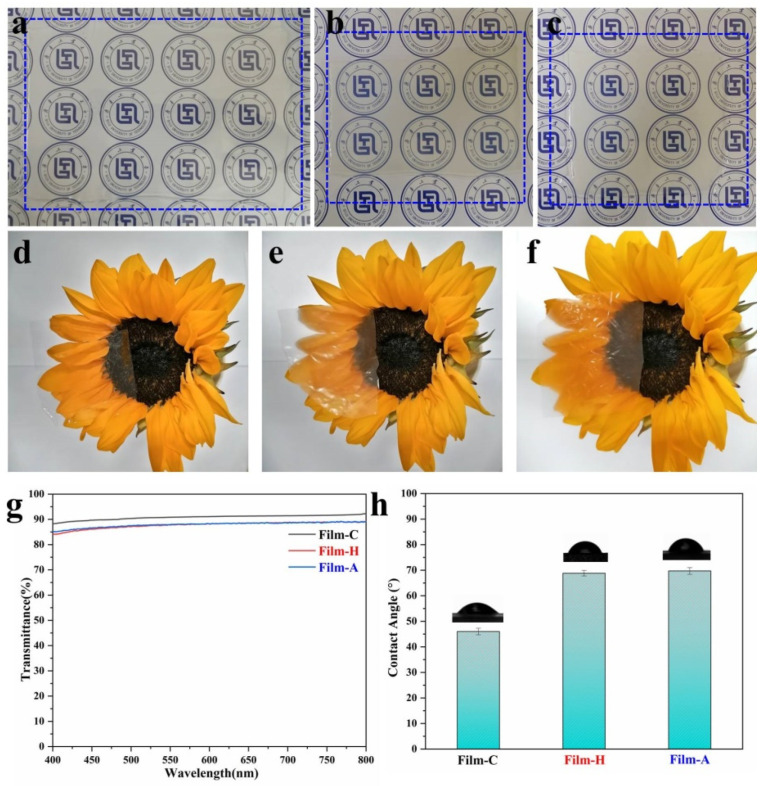
Digital pictures of (**a**) Gel-C, (**b**) Gel-H, (**c**) Gel-A, (**d**) Film-C, (**e**) Film-H and (**f**) Film-A; UV-Vis spectra (**g**) and water contact angles (**h**) of regenerated cellulose films.

The transparency of films plays an important role in their application. The digital pictures and the UV-Vis transmittance of cellulose-based materials are displayed in Figure 4a–g. It is obvious that all cellulose-based gels and films exhibited relatively good transparency and the transmittances of Film-H and Film-A at 550 nm are as high as 87.8% and 87.9%, respectively. Furthermore, the products of high grade cotton liner pulp, Gel-C and Film-C demonstrate the best transparency, which can also be quantifiably proved by the UV-Vis transmittance results (Figure 4g). Compared with that of Film-C, the transmittances of Film-H and Film-A are about 3% lower in the visible region, which may be attributed to some impurities confined in the cellulose paper board. As we know, some additives need to be added in the preparation of cellulose paper board. Additionally, most cellulose materials are hydrophilic because of copious amounts of hydrogen bonds in cellulose molecules, which severely limits their applications as packaging materials. Figure 4h demonstrates the water contact angles (WCAs) of cellulose-based film and the WCAs of C-film, Film-H and Film-A are approximately 46.0°, 68.8° and 69.7°, respectively, meaning that the cellulose-based films still exhibit good wettability. However, Film-H and Film-A possess the bigger WCAs, which can be ascribed to impurities in p-UPCs, and these impurities still exist in cellulose-based films (Film-H and Film-A). In short, cellulose-based films fabricated from the used disposable paper cups can meet the transparent demands. Moreover, compared with the traditional pure cellulose film, they display relatively good hydrophobicity, showing superiority as packaging and wrapping materials.

### 3.3. Structure and Crystallinity

**Figure 5:**
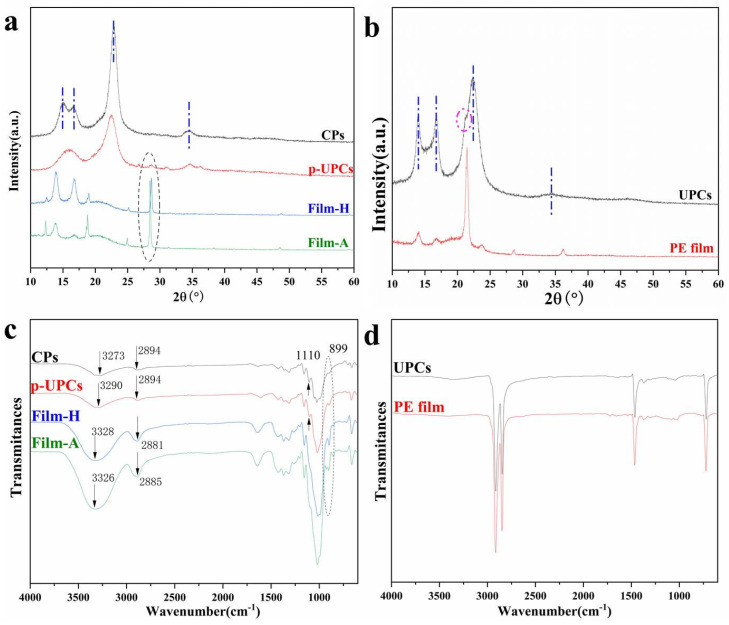
XRD of CPs, p-UPCs, W-film, and E-film (**a**) and XRD of UPCs and PE film (**b**). FTIR of CPs, p-UPCs, W-film, and E-film (**c**), and FTIR of UPCs and PE film (**d**).

The composition and crystal phases of polymer materials can be acquired by X-ray diffraction and the X-ray diffraction profiles of CPs, UPCs (the PE coating side), p-UPCs, PE film, Film-H, and Film-A were presented in Figure 5a,b. As is shown in Figure 5a, the raw material CPs, UPCs, and p-UPCs demonstrate well-defined peaks of cellulose phase I at 2θ = 15.1°, 16.8°, 22.8°, and 34.5°, corresponding to the (1–10), (110), (200) and (004) planes [12,16,31]. However, it is worth speaking that the UPCs (the PE coating side) and p-UPCs show some differences. As is presented in Figure 5b, the peaks at 13.8°, 21.6°, and 23.8° are the characteristic peaks of the PE film and the UPCs (the PE coating side) shows prominent peaks at the above positions, while these peaks were weakened for the p-UPCs, indicating that most of the PE coating can be removed after pretreatment. It is well known that there is a cellulose phase change after the dissolution and regeneration. Hence, the regenerated cellulose-based films show different profiles from that of the p-UPCs, indicating that the cellulose phase changes to II after dissolution and regeneration processes. Meanwhile, Film-H and Film-A displayed peaks at 13.8, 21.6, and 23.8°, which are attributed to the characteristic peaks of the PE coating, indicating that the p-UPCs and the regenerated cellulose-based film still contained some PE residues. The main two components of UPCs are the thin internal PE coating and cellulose paper board. In fact, it is difficult to separate the thin PE coating from the cellulose paper board efficiently and completely because the PE can permeate into the cellulose paper board voids easily during the preparation process of paper cups and PE sticks tightly to cellulose paper board. Consequently, recycling or reuse the UPCs is rare in the industry. Therefore, developing eco-friendly coating or decreasing the usage of UPCs is urgent and significant. Additionally, there is a prominent decrease in intensity for cellulose-based films compared with that of UPCs and p-UPCs, suggesting that the crystallinity index decreased a lot after dissolution and regeneration processes [12,13]. Thus, the regenerated cellulose-based films (Film-H and Film-A) display PE peaks. Meanwhile, the Film-H and Film-A showed similar profiles, meaning that the difference in crystalline structure between cellulose-based films prepared from different coagulation baths is not obvious in the XRD result of this work [15].

The change of structure and component information can be provided by the FTIR and the spectra of CPs, UPCs, p-UPCs, Film-H, Film-A, and PE film are presented in Figure 5c,d. It can be concluded that the FTIR spectra of the CPs, p-UPCs, and the cellulose-based films are almost the same, indicating that the main component of p-UPCs is cellulose and there are no chemical reactions between ionic liquid AmimCl and p-UPCs during the dissolution and regeneration processes [26,29]. In other words, the dissolution of p-UPCs in AmimCl is a physical process [12]. Nevertheless, UPCs (the PE coating side) exhibit a prominently different spectrum from those of the CPs and p-UCPs, and it is similar to the PE spectrum, meaning that the pretreatment can peel off most of the PE coating from cellulose paper board. In FTIR, some peaks such as the O-H stretching peak (3700 to 3200 cm^−1^) and C-H stretching peak (3000–2700 cm^−1^) are relatively sensitive to the cellulose structure change [12,15,16]. As is shown in Figure 5c, the O-H stretching peak is located at 3290 cm^−1^ for the raw materials p-UPCs. Nevertheless, it moves to higher wavenumbers for Film-H (3328 cm^−1^) and Film-A (3326 cm^−1^), and demonstrates an obvious blue shift. Meanwhile, the C-H stretching peak is located at 2894 cm^−1^ for p-UPCs, while it shifted to lower wavenumbers for Film-H (2881 cm^−1^) and Film-A (2885 cm^−1^), demonstrating a prominent red shift. It is worth noticing that the absorption band at 899 cm^−1^ and 1110 cm^−1^ are also usually used to analyze the changes of cellulose structure [16,28]. As is demonstrated in Figure 5c, the absorption band at 899 cm^−1^ was weak in the spectrum of CPs and p-UPCs, but it enhances obviously for Film-H and Film-A. Meanwhile, the absorption band at 1110 cm^−1^ is prominent in the spectrum of raw material CPs and p-UPCs, while it disappears in Film-H and Film-A spectra. These results suggest the differences in the hydrogen-bonding or crystal structure of cellulose after the dissolution and regeneration processes, which is shown in the XRD results.

### 3.4. Mechanical Property and Thermal Degradation

**Figure 6:**
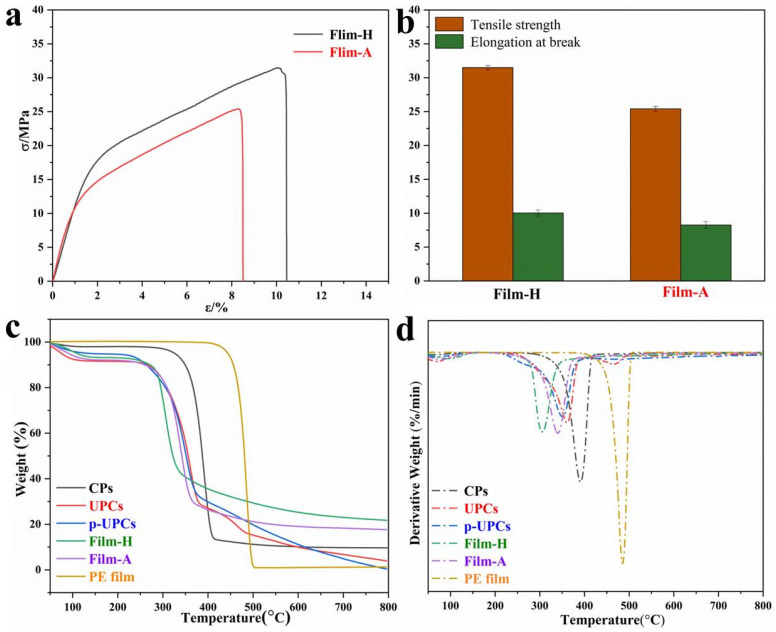
Stretch strain-stress (**a**) and tensile strength and elongation at break (**b**) of Film-H and Film-A; TG curves (**c**) and DTG curves (**d**) of CPs, UPCs, p-UPCs, Film-H, Film-A, and PE film.

Mechanical properties are the most important indexes for polymer films used as packaging materials. The stress-strain curves, the average tensile strength and elongation at break of Film-H and Film-A are shown in Figure 6a,b. Generally, the tensile strengths of polymer films directly depend on their degree of polymerization (DP) and the DP of UPCs cellulose is about 288 measured by Ubbelodhe viscometry, indicating the cellulose-based films own a relatively good mechanical property, because it is found that cellulose DP decreased slowly in AmimCl under relative mild dissolution conditions [15]. The tensile strength and elongation at the break of Film-H are 31 Mpa and 10.1%, while the tensile strength and elongation at the break of Film-H are 25.5 MPa and 8.3%, a decrease of 54.8% and 20.9%, respectively. As is presented, Film-H possesses a better mechanical property than that of Film-A, meaning that the mechanical properties of cellulose-based films are impacted obviously by the coagulation bath, which is also in correspondence with our previous studies [15,32]. By contrast, the tensile strength of the commercial PE films ranges from 9 to 12 MPa, lower than those of Film-H of Film-A [15,32]. This work demonstrates that cellulose-based films display potential applications as wrapping and packing materials. Additionally, it is common that elongation at break of the natural cellulose films is usually below 5% due to the rigid backbone structure of cellulose molecules, limiting their application [14,28]. Fortunately, the elongation at the break of cellulose-based films can be controlled by inputting plasticizers [33]. The 5% glycerol was employed to elevate the elongation at the break of cellulose-based films in this work, so the elongation at the break of Film-H of Film-A is far above 5%. Hence, preparing cellulose-based films from the used disposable paper cups is attractive and meaningful, which is helpful to tackle global climate change and meet sustainable development.

To investigate the thermal stability of the raw materials and the regenerated cellulose-based materials, TGA tests are usually employed. Both the thermogram curves (TG) and differential thermogram (DTG) curves are output to analyze the thermal stability of CPs, UPCs, p-UPCs, PE film, and regenerated cellulose-based films (Film-H and Film-A). In general, the small mass loss peak below the temperature of 200 °C is generally ascribed to the contained moisture loss in the samples [32]. As is shown in Figure 6c,d, the onset decomposition temperature (T_onset_) and temperature of maximum weight loss rates (T_max_) of CPs are 280 °C and 395 °C, higher than those of UPCs (240 °C and 360 °C), because the DP of UPCs (288 °C) is smaller than that of CPs (530 °C). It is worth noticing that UPCs display a minor DTG peak at approximately 480 °C, which is attributed to the decomposed temperature of the inner PE coating. However, the p-UPCs just have one DTG peek around 350 °C, meaning that most of the PE coating has been removed by the pretreatment. It can be concluded that UPCs displays two obvious decomposed temperature at 360 °C and 480 °C in DTG curves, ascribed to the PE and cellulose, which is common for the mechanically mixed polymer composites, because the internal PE coating and the cellulose paper board are only mechanically mixed [15]. Moreover, the T_max_ of the commercial PE film is about 485 °C, suggesting that the inner thin PE coating still possesses good thermal stability even after use. Meanwhile, the cellulose-based films (Film-H and Film-A) begin to decompose above 200 °C, due to the degradation of cellulose macromolecules [14]. As the crystallinity index of the cellulose-based films decreased sharply after the dissolution and regeneration processes, the T_onset_ and T_max_ of Film-H and Film-A are lower than that of the raw materials [14,31]. It is worth speaking that the T_max_ of Film-A (340 °C) is about 40 °C higher than that of Film-H (300 °C) due to the higher crystallinity degree of Film-A, indicating that the coagulation bath impacts the structure and thermal property of cellulose materials, which was also reported in our previous works [15]. Additionally, compared with that of p-UPCs, cellulose-based films display the higher ashes contents after 800 °C, which can be attributed to the differences of structure between the raw materials and the regenerated products.

### 3.5. Morphology of Cellulose-Based Films

**Figure 7:**
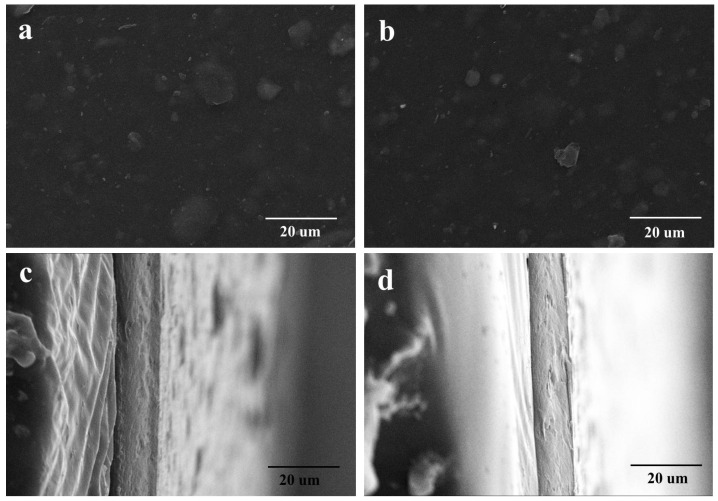
(**a**–**d**) SEM micrographs of cellulose-based films. (**a**) and (**b**) the surface images of Film-H and Film-A; (**c**) and (**d**) the cross-section images of Film-H and Film-A.

The SEM images were also recorded to characterize the morphology of cellulose-based films, as shown in Figure 7a–d. It can be seen that cellulose-based films are homogeneous and the thickness is approximately 16 um. No significant differences were found in the surface morphology for Film-H and Film-A, and they are all showing a rough surface due to some impurities. Additionally, both Film-H and Film-A possess a dense inner texture, indicating the relatively flawless of cellulose-based films, which is helpful to their mechanical property.

## 4. Conclusions

The low-cost used paper cups were successfully fabricated into high-value transparent cellulose-based films by AmimCl solvent method in this work, where the single cellulose molecular chain can be obtained by the synergistic effect of cations and anions. It was also found that cellulose transformed from I to II after the p-UPCs dissolution and regeneration processes, and some impurities were still confined in p-UPCs and the cellulose-based films, leading to that the WCAs of Film-H (68.8°) and Film-A (69.7°) were bigger than that of traditional pure cellulose film (46.0°). Meanwhile, cellulose-based films demonstrated good mechanical property and their mechanical property can be modulated by the coagulation bath. Moreover, the tensile strength and elongation at break of Film-H regenerated from deionized water are 31.5 Mpa and 10.1%, higher than those of Film-A regenerated from ethanol (25.5 MPa and 8.3%), suggesting that the coagulation bath had an important effect on the property of cellulose-based films. The flexible self-sealing bag polyethylene membrane usually employed in the laboratory had the tensile strength of about 9.0 MPa, which was quite low compared to those of cellulose-based films. Additionally, the cellulose-based films still owned good thermal property although their T_max_ and T_onset_ were lower than that of the UPCs and p-UPCs, and the Tmax of Film-H and Film-A at approximately 300 °C and 340 °C. To be concluded, the cellulose-based films produced from used paper cups can be used as wrapping and packaging materials to supplement petrochemical plastics.

Valorization of used paper cups can be achieved by fabricating high-valve transparent cellulose-based films with good mechanical property through AmimCl solvent method in this work. The low-cost used paper cups can be a good supplement for the high-grade dissolving pulp. Besides, the prepared functional cellulose-based film products show great potentials in supplementing or even replacing the non-degradable petrochemical plastics. This work is not only beneficial to the environmental protection, but also good for the maximal use of natural resources.

## Data Availability

The data presented in this study are available on request from the author.
